# Towards Efficient Milling of Multi-Cavity Aeronautical Structural Parts Considering ACO-Based Optimal Tool Feed Position and Path

**DOI:** 10.3390/mi12010088

**Published:** 2021-01-16

**Authors:** Yupeng Xin, Yuanheng Li, Wenhui Li, Gangfeng Wang

**Affiliations:** 1 College of Mechanical and Vehicle Engineering, Taiyuan University of Technology, Taiyuan 030024, China; xinyupeng@tyut.edu.cn (Y.X.); liyuanheng0039@link.tyut.edu.cn (Y.L.); 2 College of Aeronautics and Astronautics, Taiyuan University of Technology, Taiyuan 030024, China; wenhui_li7190@126.com; 3 Key Laboratory of Expressway Construction Machinery of Shaanxi Province, School of Construction Machinery, Chang’an University, Xi’an 710064, China

**Keywords:** smart manufacturing, complex structural parts, processing sequence planning, corner milling, ant colony optimization algorithm

## Abstract

Cavities are typical features in aeronautical structural parts and molds. For high-speed milling of multi-cavity parts, a reasonable processing sequence planning can significantly affect the machining accuracy and efficiency. This paper proposes an improved continuous peripheral milling method for multi-cavity based on ant colony optimization algorithm (ACO). Firstly, by analyzing the mathematical model of cavity corner milling process, the geometric center of the corner is selected as the initial tool feed position. Subsequently, the tool path is globally optimized through ant colony dissemination and pheromone perception for path solution of multi-cavity milling. With the advantages of ant colony parallel search and pheromone positive feedback, the searching efficiency of the global shortest processing path is effectively improved. Finally, the milling programming of an aeronautical structural part is taken as a sample to verify the effectiveness of the proposed methodology. Compared with zigzag milling and genetic algorithm (GA)-based peripheral milling modes in the computer aided manufacturing (CAM) software, the results show that the ACO-based methodology can shorten the milling time of a sample part by more than 13%.

## 1. Introduction

Cavity shapes in aircraft structure parts and molds are often complex with highly diverse irregularities [1]. They are usually milled at a high-speed rate by a 3-axis milling machine or a multi-axis computer numerical control (CNC) milling machine [2,3,4,5,6]. High-speed milling requires avoiding abrupt changes in the cutting direction, preventing collisions between the cutter and the part, and reducing changes in the material removal rate.

Usually, we use peripheral milling method to process a single cavity [7,8,9]. However, in the peripheral milling method, the uneven tool path will cause a sudden increase in milling force. Generally, when the arc radius of a circular milling cutter is greater than half the row distance, there will be a discontinuous connection between a straight line and an arc, which cannot meet the needs of high-speed milling. Therefore, high-speed milling needs to keep the cutting mode constant while minimizing the idle stroke of the tool. The current research on this issue mainly focuses on two aspects: (1) improving the cutting mode of a single cavity to ensure the smooth cutting process and (2) optimizing the cutting path of the tool to minimize the empty stroke of the tool.

Aiming at improving the cutting mode of a single cavity, An et al. [7] used a B-spline curve to optimize the traditional circumcision mode, but with this method it is not easy to control the cross path. Yao et al. [8] replaced the sharp corners in the circular cutting path with circular arcs, but the tool path still could not meet the requirements for smoothness of high-speed milling. Wu et al. [9] identified the tool contour path by identifying the island and cavity contour boundaries and combining the contour radius of the island contour, but the algorithm was complex and the tool path still had abrupt direction changes. Wang et al. [10] used internal interpolation polygons with linearly interpolated cavity boundaries to generate helical polylines, and B-spline curve fitting to generate continuous spiral helical tool rails of any order. However, it only solved the problem of rapid milling of cavities without islands.

To avoid cracks in the corners of cavities, secondary corner-milling is required [11]. In CNC milling the cavity, in order to avoid over-cutting or under-cutting at the corner, the programmer needs to set the arc milling program according to the transition arc radius and tool radius of the part corner [2,12], which is also defined as blend feature [13,14,15]. When the processed parts have more blend features, this method increases the difficulty of CNC programming, prolongs the time of CNC machining process programming, and affects the production efficiency of the enterprise.

In order to compensate the machining error of the part surface contour, Yue [16] proposed an automatic tool path compensation method. Karunakaran and Shringi [17] aimed at the optimized feed rate and proposed an optimization model based on NC codes. Yang [18] used the cutter location surface to improve the infeed position and optimized the tool path and processing parameters. Shi [19] improved the general hierarchical algorithm to generate milling tool paths. The verification results show that this method can keep the effective machining tool path ratio above 80%. Rao [20] optimized the peripheral milling path of variable curvature geometry by establishing a cantilever beam force model to estimate the tool deflection.

However, corner-milling is a part of cavities machining. Most researchers used the corner-milling as an independent study and rarely combined corner-milling with whole cavities tool path generation. With the development of artificial intelligence technology, researchers have tried various artificial intelligence algorithms to optimize the milling tool path of multi-cavity parts, such as genetic algorithms (GA) [7,8], ant colony optimization (ACO) algorithms [9], particle swarm algorithms [21], etc.

In order to solve the optimization problem of surface roughness during part processing, Li et al. [22] used a neural network algorithm to optimize the machining parameters and tool paths. This method manually calculates the distance between processing features and it is difficult to ensure the accuracy of the calculation results. Chu et al. [23] simplified the processing of feature boundaries in three dimensions to two-dimensional boundary curves, and then used the ACO algorithm to search the optimal solution. The division of the boundary greatly reduced the sample space, thereby greatly improving the calculation efficiency. However, the machining feature boundary of the cavity is affected by mixed features (curved surface or trapezoidal angle), which makes it difficult to obtain directly. Plakhotnik and Lauwers [24] designed a developed optimization method, which can find a series of tool positions, including the displacement of the machine’s rotating axis.

The above researchers used various methods to achieve the tool path optimization for multi-cavities. However, there are two main factors that affect the efficiency and accuracy of the calculation results. Firstly, the calculation of the spatial distance between different cavities lacks an effective method for identifying the feature position, which is mainly accomplished by manually setting the position coordinate parameters. Secondly, the interference of blend features makes the acquisition of processing boundaries inaccurate, resulting in inaccurate calculation results.

To improve milling accuracy and efficiency, in previous work, we proposed a blend feature simplification (BFS) method to accurately obtain the machining boundaries and tool feed position [25,26]. Firstly, we proposed to construct virtual boundaries of the transition feature in order to accurately obtain the tool feed position and prevent over-cutting or under-cutting. In this way, the arc surface transition feature is simplified to a linear feature. On this basis, the compensation calculation of the transition fillet radius in the corner milling programming process can be omitted. Secondly, using the coordinates of different tool feed positions at the corners, we optimized the tool paths based on genetic algorithm.

In this method, the tool feed position for a single cavity is selected randomly. In order to avoid repeatedly selecting corner tool feed positions in the same cavity, it is necessary to select a tool feed position for each cavity based on BFS. Thus, the optimization of cavities machining sequences is limited. When milling a cavity using the peripheral milling method, there are different options for the tool feed position. In theory, each corner and geometric center both can be selected. If a certain feed point is selected artificially, it is easy to cause a local optimal solution.

**Figure 1:**
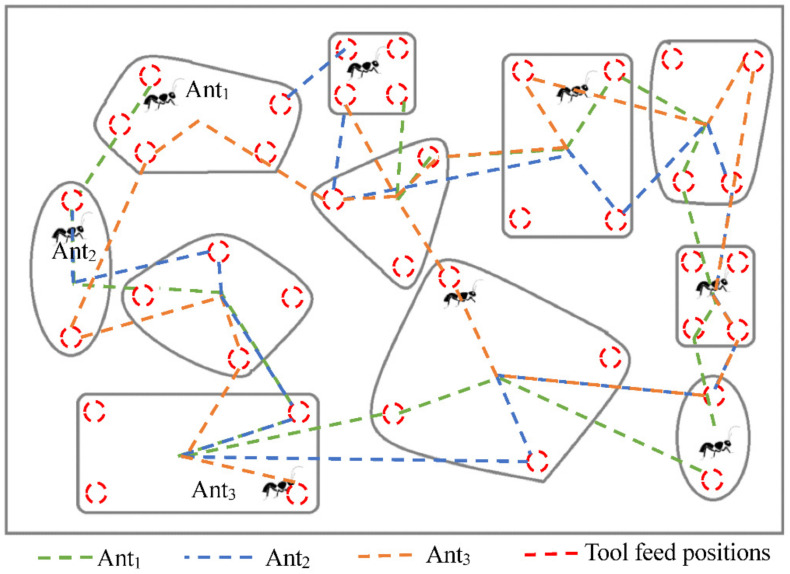
Ant colony optimization (ACO)-based tool path optimization.

Hence, this paper tries to use an ACO algorithm to make up for the shortcomings of previous work. In ACO, the pheromone released during the ant travel is used to find the shortest path. Ants with shorter paths release more pheromone. As time progresses, the concentration of pheromone accumulated on the shorter paths gradually increases, and the number of ants that choose the path increases. Finally, the entire ant colony will focus on the best path under the action of positive feedback. At the same time, it corresponds to the optimized solution of the problem. Figure 1 shows the idea of searching for the shortest processing path based on ant colony algorithm.

The remainder of this paper is organized as follows. In Section 2, we analyze the corner milling methods. Subsequently, Section 3 describes the tool path generation using the ACO algorithm. In Section 4, the feasibility of this method is proven using a tool path planning case of an aircraft structural part. Section 5 concludes the paper.

## 2. Cavity Milling Methods and Corner Milling Analysis

### 2.1. Cavity Milling Methods

**Figure 2:**
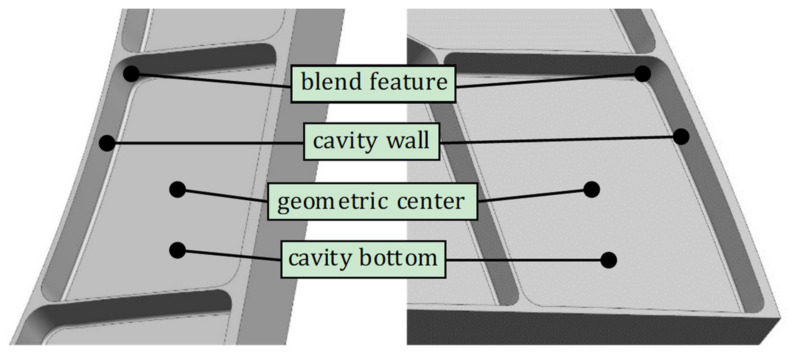
Blend features in a multi-cavity aeronautical part.

Figure 2 shows a typical aircraft multi-cavity part. Considering the overall rigidity requirements, most cavities are closed cavity. In order to avoid stress concentration at the corners, blend features are used to replace sharp edges or sharp corners.

**Figure 3:**
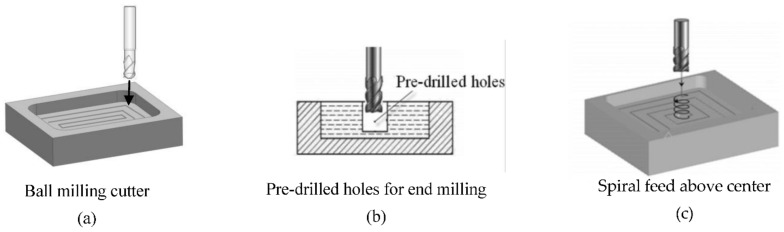
Feed methods of cavity milling.

Generally, the cavity can be processed by ball milling cutters and end milling cutters, as shown in Figure 3. The end milling cutters do not have the ability to feed in a large depth along the axial direction. Thus, for milling a closed cavity, the cutter feeds in two ways. (1) Pre-drilled holes. When rough milling of a cavity, we firstly drill a hole, which is larger than the diameter of the cutter. After the cutter reaches the cutting plane, the cavity is milled, as shown in Figure 3b. (2) Spiral feed above center. The material is milled in a spiral feed with the side and bottom cutting edges. This approach can avoid the influence of no cutting edge in the tool center [27], which is shown in Figure 3c.

**Figure 4:**
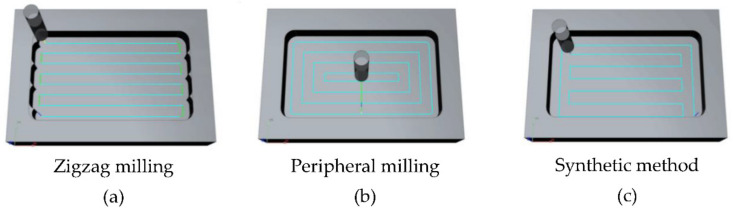
Milling methods of cavity.

When the tool reaches the milling plane to perform allowance cutting, the cutter feeds above the corner. There are three common milling methods. (1) Zigzag milling. As shown in Figure 4a, the tool travels along a zigzag path, which has a high processing efficiency. However, there will be uneven residues between two adjacent traveling tool paths, resulting in uneven finishing allowances, and the residual height is related to the tool diameter and line spacing [28,29]. (2) Peripheral milling. Figure 4b shows the tool path of peripheral milling. Using this method, the remaining allowance after rough milling is uniform. The disadvantage is the long path and low processing efficiency [30]. (3) Synthetic method. As shown in Figure 4c, the zigzag method is used for rough milling, and then the cavity is processed along the periphery. This tool path combines the advantages of the previous two methods, which is not only conducive to improving the accuracy of roughing, but also to ensuring that the machining allowance is uniform.

### 2.2. Corner Milling Analysis

When processing corners of the cavity, cutting thickness is uneven due to the sudden change in contact area between cutting tool and the part.

**Figure 5:**
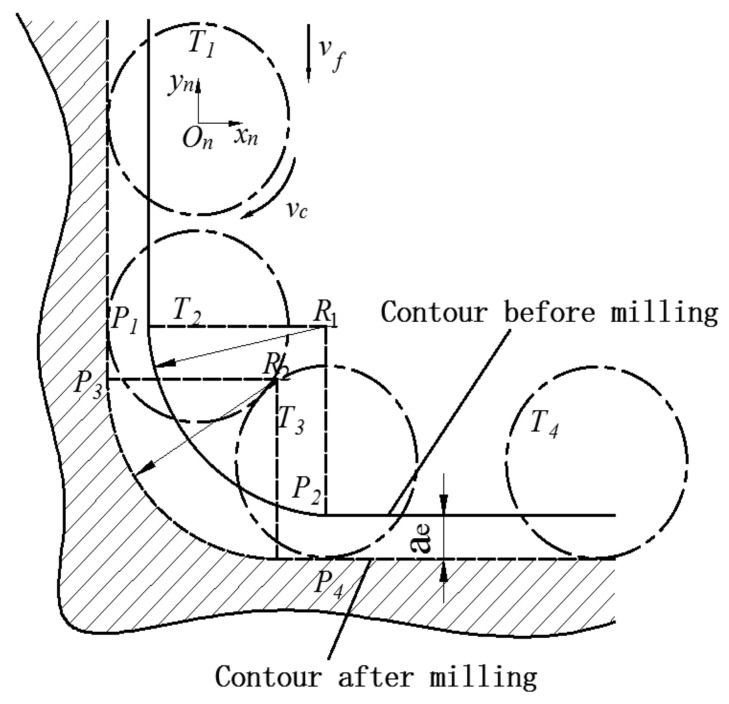
Corner milling analysis.

Therefore, in milling the corners of multi-cavity part, over-cutting or under-cutting often occurs [31]. In order to accurately design the tool path, it is necessary to grasp the change of the contact range between the tool and the part. The milling process at the corner can be divided into three stages as shown in Figure 5.

The tool path from *T*_1_ to *T*_2_ is a stable linear motion during the milling process, and a constant radial depth *a_e_* is maintained at this stage. The coordinates of the tool feed position at the corner can be obtained from the tool contour and the part contour. As shown in Figure 4, the intersection of the two contours is *P_n_*, and the coordinates of the tool feed position *O_n_*(*x_n_*, *y_n_*) can be obtained by Formulaes (1) and (2).

(1) $${\left(x-x_{n}\right)}^{2}+{\left(y-y_{n}\right)}^{2}={\left[R\left(n\right)\right]}^{2}$$

(2) $$x\;=\;\left(R\left(n\right)-a_{e}\right)-x_{n}$$

where *R_n_* is the corner radius after milling.

From *T*_2_ to *T*_3_ is the corner machining stage. The tool path is an arc, and the radial cutting depth changes from ascending to descending. We can obtain *O_n_*(*x_n_*, *y_n_*) by Formulaes (3) and (4).

(3) $${\left(x-x_{o1}\right)}^{2}+{\left(y-y_{o1}\right)}^{2}=R_{1}^{2}$$

(4) $$y=y-\left(R\left(n\right)-a_{e}\right)$$

where *O*_1_(*x*_1_, *y*_1_) is the arc center point of the corner contour before machining. The tool path after *T*_3_ becomes a stable linear motion again.

The tool feed position *O_n_*(*x_n_*, *y_n_*) can be calculated by Formulae (1) and (2). In theory, for the milling of a single cavity, the machining time for different corner infeeds is equal. However, considering the global perspective of multi-cavity part machining, choosing different corner infeeds will affect the empty travel distance from one cavity to the next, thus affecting the part processing time.

## 3. Tool Path Planning of Multi-Cavity Based on ACO Algorithm

**Figure 6:**
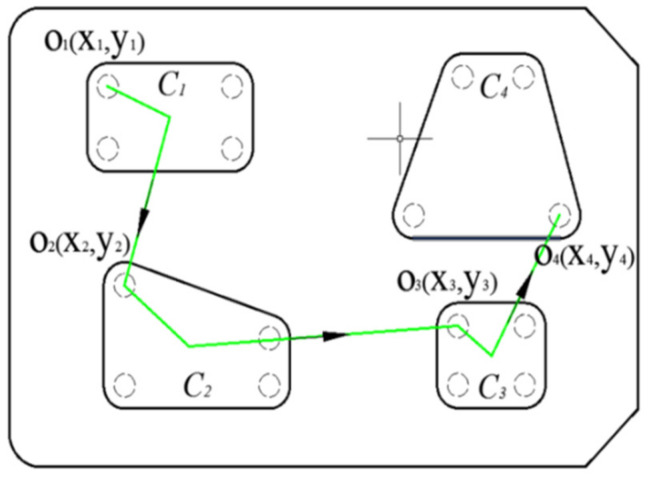
Tool path planning of a multi-cavity part.

For island-type cavities, how to choose the optimal combination of tool feed positions becomes a traveling salesman problem (TSP), as shown in Figure 6. The ACO algorithm is a probabilistic algorithm that is used to find optimized paths frequently. The algorithm constructs a solution path from the walking paths of multiple ants and improves the quality of the solution by exchanging pheromones on the solution path, thereby achieving the purpose of optimization. Compared with other heuristic algorithms, the ACO algorithm has stronger robustness and better global optimization ability.

For the problem of multi-cavity tool path planning, the influence of cavity machining sequence is significant. Therefore, we use the ACO algorithm to calculate the initial cavities machining sequence in the global scope. When the tool path is globally optimized, a unique feed position needs to be determined for each cavity. Therefore, it is necessary to add the remaining tool feed positions of the same cavity to the tabu list, which avoids obtaining repeated tool feed positions when machining the same cavity.

**Figure 7:**
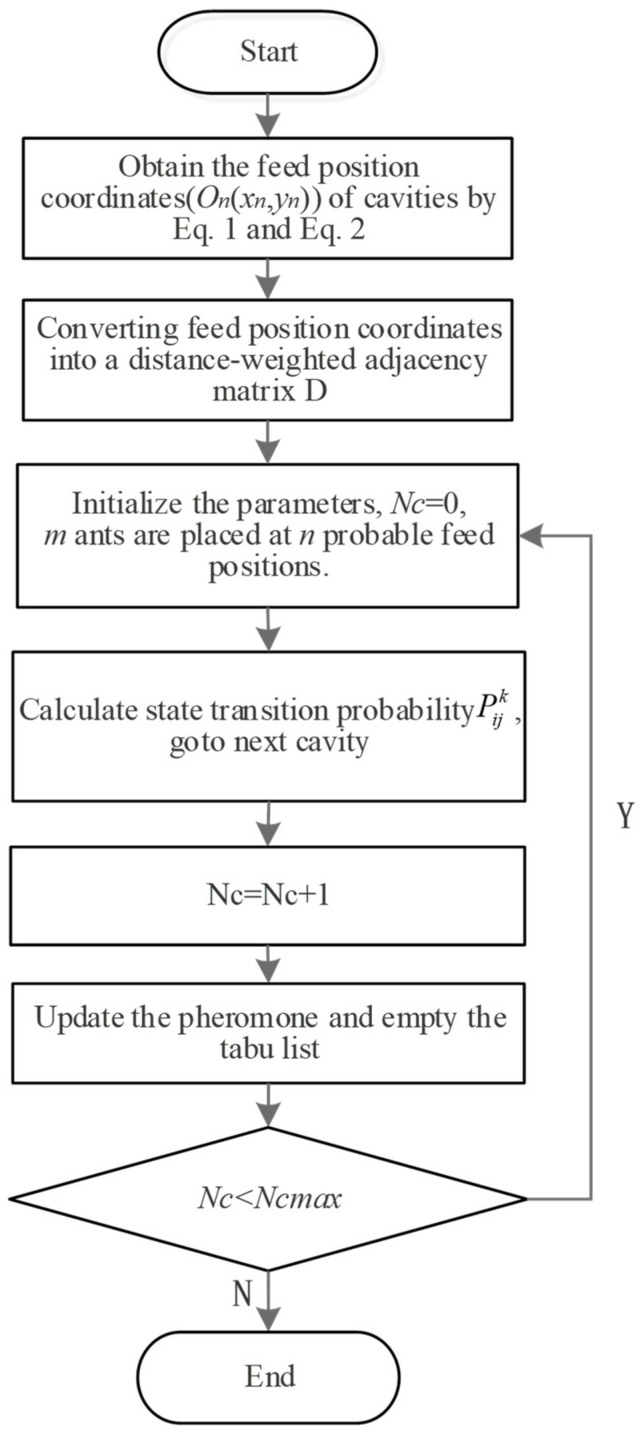
Flow chart of optimal tool feed positions using ACO algorithm.

Based on the above analysis, we designed the algorithm shown in Figure 7. The specific algorithm steps are as follows:

Step 1. Initialize all parameters. Let the number of loops *Nc* = 0 and set the maximum number of iterations *Ncmax*. Place *m* ants on *n* feed positions.

Step 2. According to the transition probability $P_{ij}^{k}$, *m* ants select the next feed position. The remaining feed positions of the same cavity are added to the tabu list, and each ant completes the travel of the respective cavity.

Step 3. Store the route distance. Let *Nc = Nc +* 1 and continue the iteration.

Step 4. Update the pheromone and empty the tabu list.

Step 5. Judge if *Nc* reaches the maximum number of iterations. If the maximum number of iterations is not reached, return to Step 1 for the next iteration, otherwise, the algorithm ends.

In Step 2, the probability $P_{ij}^{k}$ that each ant depends on when selecting the next cavity feed position is calculated according to the state transition Formula as,

(5) $$P_{ij}^{k}=\left\{\begin{array}{l} \frac{\tau_{ij}^{\alpha }\eta_{ij}^{\beta }}{\sum_{s\in {\mathrm{allowed}}_{k}}\tau_{is}^{\alpha }\eta_{is}^{\beta }},j\in {\mathrm{allowed}}_{k}\\ \;0 \end{array}\right.$$

where $P_{ij}^{k}$ is the *k*th ant’s transition probability; *j* is the feed position of unvisited cavity; $\tau_{ij}^{k}$ is the pheromone track strength of $path\;\left(i,j\right)$; $\eta_{ij}^{k}$ is visibility of $path\;\left(i,j\right)$, which reflects the inspiration level of transition; α is information heuristic factor, indicating the relative importance of accumulated information; β is expected heuristic factor, indicating the relative importance of visibility; allowed*_k_* is the position of tool feed that each ant can choose after one transfer. The range of allowed*_k_* is ${\mathrm{allowed}}_{k}=\left\{0,1,\ldots ,n-1\right\}$.

In order to meet the needs of all ants passing through all cavities, it is necessary to create a data structure called tabu list to store the tool feed positions that the ants have passed. Since there may be more than one feed position for each cavity, the ants should exclude the remaining feed positions of the same cavity before proceeding to the next path selection. In order to avoid the residual information flooding the heuristic information caused by large amounts of residual pheromone, after each ant finishes a cavity or completes the traversal of all cavities (that is, the end of one cycle), the residual information is updated. The amount of pheromone on each path can be modified as

(6) $$\tau_{ij}\left(t+1\right)=\rho \cdot \tau_{ij}\left(t\right)+\Delta \tau_{ij}\left(t,t+1\right)$$

(7) $$\Delta \tau_{ij}\left(t,t+1\right)\;=\overset{m}{\underset{k=1}{\sum }}\Delta \tau_{ij}^{k}\left(t,t+1\right)$$

where $\Delta \tau_{ij}^{k}\left(t,t+1\right)$ is the pheromone left by *k*th ant at the $time\;\left(t,t+1\right)$ on the $path\;\left(i,j\right)$. $\Delta \tau_{ij}^{k}\left(t,t+1\right)$ means the increment of the pheromone quantity of the $path\;\left(i,j\right)$ in this loop. ρ represents the residual coefficient of the pheromone.

In general, $\Delta \tau_{ij}^{k}$ has three pheromone update models which include ant-quantity model, ant-density model, and ant-cycle model. The ant-cycle model uses global information, that is, the ant updates the pheromone on all paths after completing a loop, and the ant-quantity and ant-density models use local information, that is, the ant updates the pheromone on the path after completing one step [23]. Therefore, we use the ant-cycle model for the global update of pheromone in multi-cavity tool path planning. The value of updated pheromone can be obtained as

(8) $$\Delta \tau_{ij}\left(t+n\right)=\left\{\begin{array}{l} \frac{Q}{L_{k}},\;\;\;\mathrm{ant}\;\mathrm{is}\;\mathrm{in}\;\mathrm{the}\;\mathrm{path}\left(i,j\right)\\ 0,\;\;\;\mathrm{ant}\;\mathrm{is}\;\mathrm{out}\;\mathrm{of}\;\mathrm{the}\;\mathrm{path}\left(i,j\right) \end{array}\right.$$

where $L_{k}$ is the length of the path taken by the *k*th ant in this cycle.

## 4. Implementation and Verification

In order to verify the effectiveness of the proposed method, the CNC tool path planning of an aircraft structural part (shown in Table 1) was used as a sample. This part mainly involves 20 cavities, and each cavity corner adopts blend features which should be considered compensation in CNC programming.

### 4.1. Obtain the Coordinates of Tool Feed Positions

**Figure 8:**
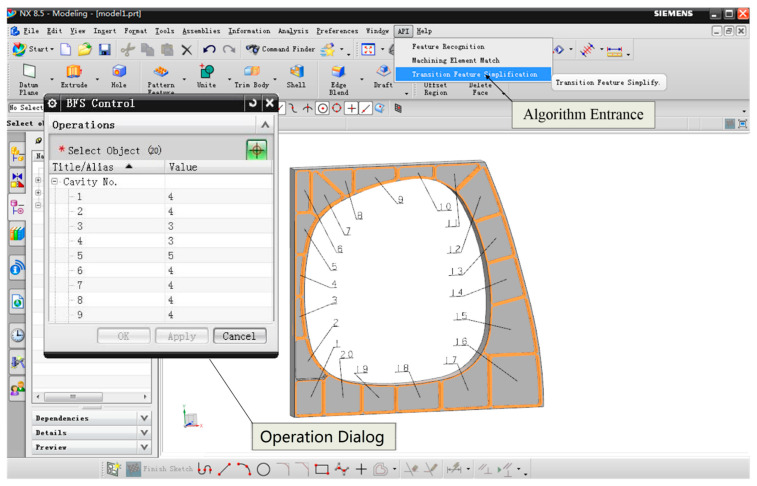
The alternative tool feed positions of each cavity.

In this case, the tool feed positions of cavities are obtained by Formulaes (1) and (2). We make use of the application programming interface of the Siemens Unigraphics (UG) software and carry on the secondary development to UG, which realizes the algorithm, as shown in Figure 8.

Combined with the 3D model as shown in Figure 8, part of the tool feed positions coordinates are shown in Table 2. There is a total of 85 tool feed positions in all the 20 cavities, which form a coordinate position matrix *P*.

### 4.2. Optimization of Milling Tool Path Using ACO Algorithm

The number of ants is determined by the number of cavities. Combined with Table 3, a total of 20 ants were randomly assigned to 85 tool feed positions in 20 cavities to construct an initial random distribution. In the TSP optimization process of the same scale as sample, the optimal range of information heuristic factor *α* is [0.7, 1.1], the expected heuristic factor *β* has a value range of [3.8, 4.5], the pheromone intensity *Q* value can be selected between 1 and 20, the residual coefficient of the pheromone *ρ* has little effect on time, when *ρ* = 0.5, the optimal solution performance is better [23]. According to Section 4, the distance-weighted adjacency matrix *P* is obtained from the tool feed position matrix *D*. The control parameters and algorithm codes are shown in Table 3. In the algorithm codes, each ant selects the next corner according to the probability calculated by Formula (5). Once the corner feature of the same cavity is selected, the remaining tool feed positions of this cavity are added to the tabu list and are no longer candidates. Each ant stops moving as it passes through all the cavities.

**Figure 9:**
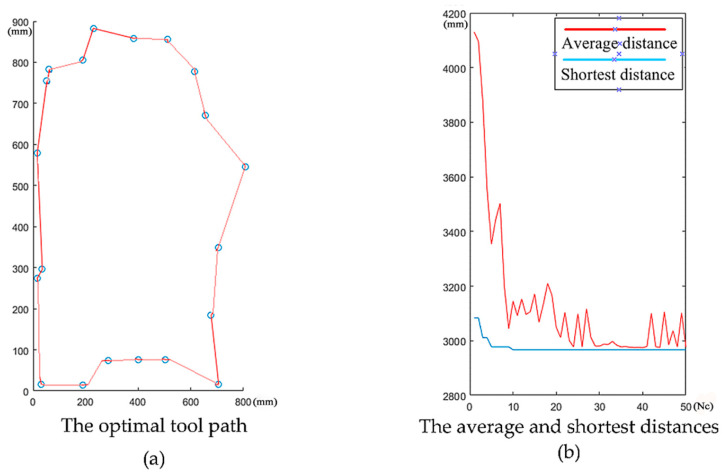
The calculation results shown in Matlab.

We record the moving length of each ant and select the shortest ones. Then, the pheromone is updated and the pheromone information on the path (considering the pheromone accumulation and volatilization for each movement) is stored to the pheromone matrix. In the end, we empty the tabu list for the next iteration. When the number of iterations reaches the set value, the iteration is stopped and the resulting optimal path is returned. For the purpose of the validity of the algorithm, we implemented a verification experiment in Matlab. The results are shown in Figure 9. In this experiment, the terminate condition (maximum iterations *Nc*) is set to 50. As shown in Figure 9a, we can obtain the optimal tool path using ACO algorithm from 85 tool feed positions of 20 cavities. In Figure 9b, we can see that the shortest distance tends to stabilize after 10 iterations and the average distance traveled by the ants tends to be stable at about 3000 mm. This implies that the tool moving distance at 3000 mm, the machining time may be the shortest.

### 4.3. Comparative Analysis of Simulation and Experiment Results

**Figure 10:**
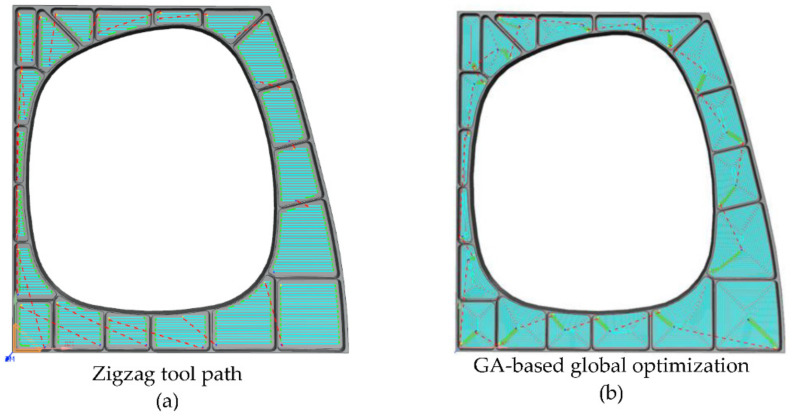
Tool paths simulation in the Siemens Unigraphics (UG) software.

**Figure 11:**
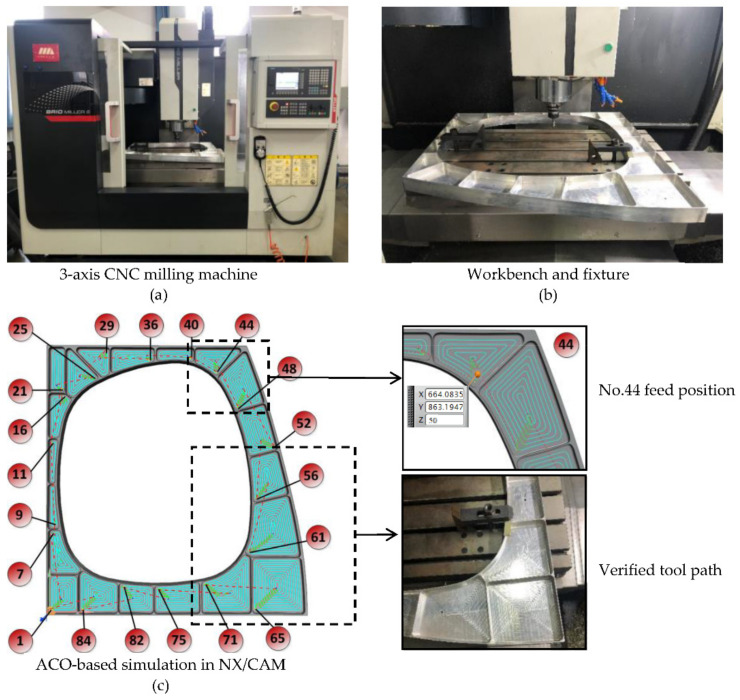
ACO-based simulation path and machining experiment.

In order to verify the advantages of the method proposed in this paper, we performed a comparative simulation experiment in the UG software. The ball end milling cutter we used has a diameter of 10 mm and a length of 75 mm. The main process parameters are given in Table 1. In order to achieve the comparison verification, we simulate three different tool paths in the UG software, as shown in Figure 10 and Figure 11.

In Figure 10a, the first tool path follows zigzag. To compare the efficiency of different milling methods, Figure 10b shows the tool path of peripheral milling, and the idle strokes between cavities are optimized by improved-GA algorithm, which was described in detail in previous work [25]. Figure 11 shows the simulation path and machining experiment based on ACO algorithm. Table 4 shows the machining time required to use these three tool paths.

The Siemens SINUMERIK 808D CNC system is adopted in the machining experiment. The G codes of tool path shown in Figure 11c are generated by the the UG software post-processing function. The tool is a ball milling cutter (Φ12 mm × 30 mm × 60 mm), and the milling parameters are shown in Table 1. The Perthometer M2 was used to measure the four walls of the No. 19 cavity. The measurement results, as shown in Table 5, represent that the processing has good uniformity and the surface roughness meets the requirements, which verifies the validity of the simulation trajectory.

Based on the above experiments, we obtain the following results:

- As shown in Figure 9b, we can see that the shortest distance tends to stabilize after 10 iterations. This proves the superiority of the ACO algorithm in multi-cavity milling path optimization.
- In Figure 11c, a total of 20 tool feed positions were obtained by ACO algorithm. On this basis, it is more suitable for automatically calculating the distance of tool paths. We can get the optimized machining sequence of the 20 cavities.
- Table 4 shows that the machining time of the ACO-based optimal tool path is 21 min shorter than the zigzag milling path and is 14 min shorter than GA-based peripheral milling. Computational results show that the tool path planning method proposed in this paper can effectively shorten the machining time.
- The machining results in Table 5 show that the milling method mentioned in this article can guarantee high quality machining uniformity.

## 5. Conclusions and Future Work

This paper proposed and validated a novel NC tool path planning method for high-speed milling of multi-cavity part based on ACO algorithm. Some of the main contributions of this research are listed below.

- For the milling of inner cavity corners by peripheral milling method, we established a mathematical model of the corner milling process. Based on this, we combine with ACO algorithm to select the optimal tool feed position for each cavity.
- The ACO algorithm is used to optimize the tool path of multi-cavity part. Compared with the other two commonly used tool path planning methods, the method proposed in this paper can shorten the machining time by more than 13%.

In the near future, several issues are worth being further researched: (1) extension of our research to other machining features, e.g., optimization of process routing in machining a group of holes, and (2) exploration the automatic identification technology of blend features to support intelligent NC machining process planning.

## Figures and Tables

**Table 1:** The dimension and process parameters of sample part.

**Dimension**				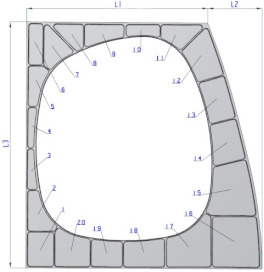
Top border L1 (mm)	669.2	Bottom border L2 (mm)	869.5	
Height L3 (mm)	910.8	Deep Dc (mm)	23	
Thickness D (mm)	50	Material	7B04-T651	
**Process Parameters**				
*Vc* (r/min)	2000	ap (mm/min)	0.5	
*Vf* (mm/min)	250	Tool material	D16	

**Table 2:** Tool feed position coordinate (cavity 1–cavity 5).

Cavity	No.	Coordinate			Cavity	No.	Coordinate		
		x	y	z			x	y	z
1	①	92.60	13.98	50	4	⑫	20.87	426.45	50
	②	15.84	14.47	50		⑬	35.12	295.79	50
	③	90.56	119.07	50		⑭	14.14	448.01	50
	④	17.81	119.27	50	5	⑮	15.89	579.60	50
2	⑤	40.32	276.37	50		⑯	21.51	449.41	50
	⑥	15.50	273.91	50		⑰	33.45	578.54	50
	⑦	17.98	146.72	50		⑱	17.30	602.75	50
	⑧	105.59	152.34	50		⑲	20.73	730.61	50
3	⑨	99.56	144.64	50					
	⑩	14.82	297.29	50					
	⑪	15.60	426.99	50					

**Table 3:** Control parameters and codes of ACO algorithm.

Parameters	*m*	*α*	*β*	*Q*	*ρ*	*Nc*
**Value**	20	0.7	3.8	20	0.5	50
**Code**	**Input** P, Nc, m **Output** Optimal tool path 1: for *j* = 2:*n* 2: for *i* = 1:*m* 3: visited = Tabu(*i*,1:(*j* − 1)); 4: *J* = zeros(1,(*n* – *j* + 1)); 5: *P* = *J*; 6: *Jc* = 1; 7: for *k* = 1:*n* 8: **if** length(find(visited == *k*)) == 0 9: **if** length(find(Tabu(*i*,1:*j*) == *C*(*k*,4))) == 0 10: *J*(*Jc*) = *k*; 11: *Jc* = *Jc* + 1; 12: **end** 13: **end** 14: **end** 15: for *k* = 1:length(*J*) 16: *P*(*k*) = (Tabu(visited(end),*J*(*k*))^Alpha)*(Eta(visited(end),*J*(*k*))^Beta); 17: **end** 18: *P* = *P*/(sum(*P*)); 19: Pcum = cumsum(*P*); 20: Select=find(Pcum ≥ rand); 21: to_visit = *J*(Select(1)); 22: Tabu(*i*,*j*) = to_visit; 23: **end** 24: **end**					

**Table 4:** Machining time comparison of three tool paths.

Tool Path Type	Machining Time (h/min/s)
Zigzag	02:00:30
Peripheral	01:54:09
ACO-based tool path	01:39:15

**Table 5:** Surface roughness measured of the No. 19 cavity.

Measurement Platform	The No. 19 Cavity	
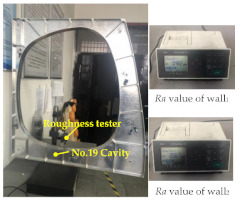	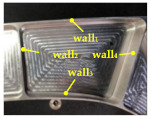	
	The measurement data (μm)	
	wall_1_	wall_2_
	0.482	0.567
	wall_3_	wall_4_
	0.503	0.512

## Data Availability

No new data were created or analyzed in this study. Data sharing is not applicable to this article.
